# Dr. C. Sedillot on Amputations

**Published:** 1850-03

**Authors:** 


### *Dr. C. Sedillot on Amputations.*

**To the Editor of the Boston Medical Journal:**

Sir:—I send you these little translations from the "Comptes Rendus," with the view of drawing attention to the subjects of which they treat. The statistics of the amputations given by Sedillot, if nothing else, are useful, and will assist in forming an opinion of the relative value of amputations. The second article is worthy of our attention at the present time, as the curriculum of ligature, according to the Hunterian method, and pressure, has been rapidly running through in surgical practice, during the last few years. The old and favorite operation of Hunter was thrown entirely into the shade, by the furor in favor of pressure, as a means of curing aneurisms so ably advocated by Liston, and his compeers the British and Irish surgeons. Pressure has gained but little ground in Philadelphia; and generally in the United States, has not obtained that confidence, which it did in England and Ireland.

This article will perhaps turn attention to the methods of the old fathers in surgery; for there seems to be in medicine, as in other things, an occasional recurrence to *old things*. Yours very truly,

*Philadelphia, Jan. 23d,* 1850. James Bryan.

Dr. Sedillot, of Strasburg, presented a memoir on the amputations of the extremities, in which he stated some new results obtained since his last communication on the same subject.

I have established (says he) the possibility of saving a greater number of patients, by a method, whose superiority both theory and practice demonstrate, and I have recited twelve cases of amputation in which I have lost but one patient. This success is remarkable in view of the terrible mortality which continually follows amputations in our hospitals; but it may be accidental, and further observations are necessary.

I performed ten amputations during the academic year 1848–9, as follows.—two of the thigh, two of the leg, one of the arm, two of the fore-arm, a partial one of the foot, and two of the phalanges. I lost but one patient, the foot amputation—which resulted in purulent infection and death. In recapitulating the results of these two years, I find

| | | | | | | |
|---|---|---|---|---|---|---|
| 3 | amputations of the | Thigh, | cured | 3, | died | 0 |
| 8 | " | Leg, | " | 8, | " | 0 |
| 2 | " | Arm, | " | 2, | " | 0 |
| 3 | " | Fore-arm, | " | 3, | " | 0 |
| 2 | " | Hand, | " | 3, | " | 0 |
| 3 | " | Foot, | " | 1, | " | 2 |
| — | | | | — | | — |
| 21 | | | | 20 | | 2 |

This general mortality was thus; 1 in 11, which fell upon those of the feet alone. We believe that with greater faith in the resources of our art, and by resorting to a secondary amputation to arrest the purulent infection, as we have advised in our "Treatise on Purulent Infections," these cases might have been saved.

If we compare amputations in continuity with those in contiguity, we find the latter much more serious—as they furnished two deaths in six cases operated upon, while the first give six cures to one death. We find also a great superiority in secondary or consecutive amputations, whose mortality was one in nineteen, while that of the primitive amputations was one third.

Dr. Sedillot presented a second memoir whose title was—"On the Section of the Arteries, in the interval of two Ligatures," as a general method of treating hæmorrhages aneurisms.

The ligation of arteries remains one of the most serious operations in surgery, in spite of the great labor undergone in order to diminish its danger.

I propose, says Sedillot, to myself an ungrateful and difficult task, to revive a method of operating, improperly abandoned. Celsus first spoke of this operation, and Ætius did but quote from Galen, in recommending it two or three centuries afterwards. From this time the section of arteries between two ligatures has not been entirely neglected. The Arabs had recourse to it. Dionis, J. Bell, J. P. Maunoir, A. Cooper, Cline, Abernethy, and many other distinguished surgeons, have sanctioned the operation.

This method is, however, at the present day, almost entirely abandoned, and Sedillot thinks without sufficient cause. He attributes the temporary prejudice to the great influence of Scarpa, who blamed the operation of Celsus, and replaced it by a very ancient process, which he supposed was superior. In our day, Roux, with whom Sedillot regrets on this point not to agree in opinion, continues alone to follow the example of Scarpa.

Velpeau is quite opposed to the plan of Celsus, and combats it with the three-fold authority of his teachings, his practice, and his writings.

Sedillot, in the face of such distinguished opponents, examined the question with great care, and arrives at the conclusion, that the series of objections presented by Velpeau are not as important as his distinguished colleague supposes.

The retractility of the arteries has been denied, and the authority of Beclard quoted! This author has on the contrary formally recognized the retractile power of the arteries, and Sedillot has pointed out the extent and condition of this retractility.

The throwing off of the ligatures by the impulse of the blood, has been admitted. Sedillot proves, by a series of experiments on this subject, that a ligature of any waxed thread, tied with two knots, as is generally done, is *never* moved by the action of a fluid, driven with force enough to rupture the artery.

Sedillot has also subjected an arterial ligature to more than the pressure of one atmosphere by a column of mercury. A ligature, placed in water to avoid desiccation, resisted, until the artery from softening gave way beyond it.

Sedillot's memoir is divided into two parts, theoretical and practical.

In the first, the author has sketched the history of the operation, and studied its advantages under three principal heads: —

1st. The superiority of a method whose execution is least exposed to the faults and errors of operators.

2d. The less frequency of hæmorrhage.

3d. The greater facility of remedying this accident when it has occurred.

The objections then follow in review, and the indications and contra-indications, with the operative portion, form as many particular chapters.

The second part of the memoir narrates eleven cases of arteries tied by Sedillot according to the rules of his method, with complete success. Twelve other cases, where results were equally happy, are selected from Cooper, Cline, Abernethy, and Maunoir of Geneva.

Examples of fatal hæmorrhage after the operation of Celsus, appear to have been very rare; for Sedillot has found but two cases reported by Lisfranc, who does not give his authority. Sedillot does not consider the question as decided, by the numerous successful cases which he publishes. Surgical reasoning is in general too complex, to allow of absolute deductions: but in examining the facts, both by reason and experience, he thinks that there is reason why we should return to an operation, against which there is no legitimate objection, and whose advantages are confirmed by a long series of ages.

